# Urban air pollution and chronic respiratory diseases in adults: insights from a cross-sectional study

**DOI:** 10.3389/fpubh.2025.1507882

**Published:** 2025-03-12

**Authors:** Mohammed M. Alyami, Fahad H. Balharith, Sunil Kumar Ravi, Ravi Shankar Reddy

**Affiliations:** ^1^Respiratory Therapy Department, Batterjee Medical College, Khamis Mushait, Saudi Arabia; ^2^Department of Medical Rehabilitation Sciences, College of Applied Medical Sciences, King Khalid University, Abha, Saudi Arabia

**Keywords:** air pollution, respiratory health, chronic respiratory conditions, spirometry, urban population

## Abstract

**Objectives:**

Air pollution poses a substantial public health risk, especially in urban environments characterized by elevated levels of pollutants such as fine particulate matter (PM2.5) and nitrogen dioxide (NO2). These pollutants negatively impact respiratory health, contributing to chronic respiratory conditions and reduced lung function. This study investigated the association between air pollution exposure and respiratory health outcomes, including the prevalence of chronic respiratory conditions and pulmonary function, in an urban population. Additionally, the study sought to identify demographic subgroups that are unprotected from the ill effects of air pollution.

**Methods:**

A cross-sectional study included a total of 1,300 adult participants recruited from outpatient pulmonary and general medicine clinics. Air pollution exposure was assessed based on residential proximity to major traffic routes and ambient levels of PM2.5 and NO2 obtained from the Riyadh air quality monitoring network. Lung function was measured using spirometry, and data on chronic respiratory conditions were collected through self-reports and medical record reviews. Multivariable logistic regression and linear regression models were used to analyze the association between air pollution exposure and respiratory outcomes, adjusting for confounders such as age, gender, smoking Status, socioeconomic Status, physical activity, and occupational exposures.

**Results:**

Higher air pollution level exposures were significantly associated with an increased prevalence of chronic respiratory conditions (adjusted OR for high exposure: 2.45, 95% CI: 1.70–3.55, *p* < 0.001) and a reduction in lung function, as indicated by declines in FEV1 and FVC (adjusted FEV1 coefficient for high exposure: -0.45, 95% CI: −0.58 to −0.32, *p* < 0.001). Subgroup analyses revealed that older adults, males, and current smokers were particularly susceptible to the adverse effects of air pollution. Sensitivity analyses confirmed the robustness of these findings across different analytical scenarios.

**Conclusion:**

The study demonstrated a clear and significant association between higher air pollution level exposures and an increased risk of chronic respiratory conditions and reduced lung function. These findings highlight the need for specific interventions to decrease air pollution exposure, particularly in vulnerable urban populations, to mitigate the burden of respiratory diseases.

## Introduction

Air pollution remains a significant global public health concern, supported by mounting evidence linking it to various adverse health outcomes (1). The expansion of urban areas, industrial activities, and the proliferation of motor vehicles have all contributed to heightened ambient air pollution levels, especially in densely populated urban centers (2). Key pollutants like particulate matter (PM), sulfur dioxide (SO2), nitrogen dioxide (NO2), and ozone (O3) are prevalent in these settings and extensively researched for their health impacts (3). Fine particulate matter (PM2.5) can reach the deeper lung regions, triggering inflammatory responses, while ultrafine particles (PM0.1) have the potential to translocate into the bloodstream (4). Epidemiological studies consistently demonstrate that prolonged exposure to these pollutants is linked to increased morbidity and mortality, notably from cardiovascular and respiratory diseases (4). Recognizing air pollution as a paramount factor for global disease burden risk, the World Health Organization (WHO) underscores the critical imperative to address this pressing environmental challenge (3).

Assessing respiratory health through lung function analysis is crucial in understanding the impact of air pollution (5). Spiro-metric measurements, such as forced expiratory volume in 1 s (FEV1) and forced vital capacity (FVC), serve as key indicators of pulmonary function and play a critical role in diagnosing and monitoring respiratory illnesses like asthma, chronic obstructive pulmonary disease (COPD), and chronic bronchitis (6). Decreases in FEV1 and FVC signal impaired lung function, often attributable to prolonged exposure to environmental pollutants (6). Studies have consistently shown that people residing in areas with elevated air pollution levels experience significant declines in lung function, independent of factors like smoking and occupational hazards (6). These declines not only reflect respiratory impairment but also predict adverse long-term health outcomes, including heightened risks of hospitalization and premature mortality (7). Monitoring and mitigating environmental exposures are therefore essential to safeguarding respiratory health in the face of air pollution challenges (8).

Subgroup analysis is an essential methodological approach in epidemiological studies, allowing researchers to identify populations that are susceptible to the ill effects of air pollution (9). Factors such as age, gender, smoking Status, and pre-existing health conditions can influence an individual’s susceptibility to air pollutants (10). Older adults may experience more severe health effects due to age-related declines in physiological resilience, while smokers may face compounded risks due to the combined effects of tobacco smoke and ambient air pollution (11). By conducting subgroup analyses, studies can provide insights into how different segments of the population are affected by environmental exposures, enabling the development of targeted public health interventions (12). Sensitivity analysis further enhances the robustness of study findings by testing the stability of results under various assumptions and methodological scenarios (13). This approach helps to ensure that observed associations are not artifacts of specific analytical choices and that the results are generalizable to broader populations (13).

Despite the growing body of literature on air pollution and respiratory health, several research gaps remain. Much of the existing research has focused on short-term exposure and acute health effects, with less emphasis on long-term exposure and chronic conditions (14). Furthermore, while numerous studies have demonstrated the overall impact of air pollution on lung function, few have systematically examined the differential effects across various demographic and clinical subgroups (15). Understanding how factors such as age, gender, and smoking status amplify the association between air pollution and respiratory health is crucial for developing effective public health strategies (16). Additionally, previous studies have often relied on aggregate measures of pollution exposure, which may not accurately reflect individual-level exposures (17). More refined analyses are needed that consider variations in exposure levels within populations and across different environmental settings. Addressing these gaps will enhance our understanding of the complex interactions between air pollution and respiratory health and inform more effective policies to mitigate the adverse effects of environmental pollutants.

The present study aims to address these gaps by systematically examining the association between air pollution exposure and respiratory health outcomes in a large urban population. The study has four primary objectives: (1) to find the association between air pollution exposure and the prevalence of chronic respiratory conditions, (2) to study the effect of air pollution on lung function as measured by FEV1 and FVC, (3) to conduct subgroup analyses to identify populations that are at risk to the ill effects of air pollution, and (4) to perform sensitivity analyses to test the robustness of the observed associations across different analytical scenarios.

## Materials and methods

### Design

This cross-sectional study was carried out from April 2022 to February 2023 at a tertiary hospital in Riyadh, Saudi Arabia, and examined the relationship between air pollution exposure and respiratory health outcomes. Participants were recruited from outpatient pulmonary and general medicine clinics, with air pollution exposure assessed based on residential proximity to major traffic routes and ambient pollutant levels. Lung function was measured using spirometry, and demographic details and clinical data were collected through interviews and medical records. Ethical approval was granted for the study by DRS, KKU (REC#345–2022) on 23/03/2022, and written informed consents were obtained from all the subjects.

### Participants

The study included a total of 1,300 adult participants recruited from the outpatient pulmonary and general medicine clinics in Riyadh, Saudi Arabia. Eligible participants were adults aged 18 years and older who had resided in Riyadh for a minimum of 5 years, ensuring sufficient exposure to the city’s air pollution levels for meaningful analysis. Participants were required to be able to give informed consent and complete the study procedures, including spirometry testing and interviews. Exclusion criteria included individuals with acute respiratory infections at the time of assessment, as these could temporarily affect lung function measurements. Participants with known interstitial lung disease, those who had undergone lung surgery, or individuals on long-term oxygen therapy were also excluded to avoid confounding factors that could influence respiratory health outcomes independently of air pollution exposure. The selection process aimed to include a diverse sample of the urban population, reflecting varying levels of air pollution exposure due to differences in residential proximity to major traffic routes and environmental settings within the city.

### Variables and data collection

The primary outcome variables in this study were chronic respiratory conditions and lung function parameters. Chronic respiratory conditions include asthma, chronic bronchitis, and COPD. These conditions were identified through a combination of self-reported medical history and confirmation via a review of participants’ medical records from King Khalid University Hospital. Participants were queried about previous diagnoses of respiratory diseases, and these responses were cross-verified with their clinical records to ensure accuracy and reliability in diagnosing chronic respiratory conditions.

Lung function was assessed using spirometry, with FEV1 and forced vital capacity (FVC) serving as the key measures. Spirometry tests were conducted according to the American Thoracic Society (ATS) guidelines, ensuring standardized procedures across all participants. Each participant performed the test a minimum of three times to ensure reproducibility and accuracy, with the highest values of FEV1 and FVC recorded for subsequent analysis. These spirometric measurements provided a quantitative assessment of lung function, allowing for the evaluation of any declines that may be associated with varying levels of air pollution exposure.

The primary exposure variable was air pollution, specifically focusing on the levels of fine particulate matter (PM2.5) and nitrogen dioxide (NO2). Data on air pollution levels were obtained from the Riyadh air quality monitoring network, which provided continuous measurements of pollutant concentrations at various locations throughout the city. Pollutant levels were recorded throughout the study period at regular intervals for comprehensive coverage. Participants’ residential addresses were geocoded and linked to the nearest monitoring station, with proximity to major traffic routes calculated using GIS software. Exposure was categorized into three thresholds: <1 km, 1–3 km, and > 3 km, with closer distances indicating higher pollution exposure. Mean annual PM2.5 and NO2 concentrations were used to assess long-term exposure, minimizing short-term variability and aligning with chronic exposure research. Distance classifications were chosen based on Riyadh’s urban layout, residential patterns, and monitoring station distribution. While some studies apply shorter distances (100 m, 250 m, 800 m) for traffic-related pollution assessment, our approach ensures effective differentiation within the city’s spatial framework.

Exposure classification was based on a composite measure integrating residential proximity to major traffic routes and annual mean concentrations of NO2 and PM2.5. Low exposure was defined as residing >3 km from major roads with NO2 < 35 μg/m^3^ and PM2.5 < 20 μg/m^3^; moderate exposure included those residing 1–3 km from major roads with NO2 levels of 35–50 μg/m^3^ and PM2.5 of 20–30 μg/m^3^; and high exposure included individuals living <1 km from major roads with NO2 > 50 μg/m^3^ and PM2.5 > 30 μg/m^3^. Sensitivity analyses were conducted by separately assessing pollutant concentrations and traffic proximity to enhance comparability with existing studies.

Demographic variables included age and BMI as continuous measures, while gender, marital status, and residential Area were categorical. Smoking status was classified as current, former, or never smoker based on self-reports. Socioeconomic Status was determined through a structured questionnaire assessing education level, household income, and occupation, categorizing participants into low, middle, or high groups. Occupational exposure was evaluated based on job type and exposure to respiratory hazards, distinguishing manual from non-manual workers. Physical activity levels were categorized as low, moderate, or high using a standardized questionnaire assessing weekly exercise frequency and intensity. Pre-existing chronic conditions were self-reported and verified through medical records. Structured survey questions provided standardized assessments of smoking history, occupational exposure, and physical activity based on validated epidemiological criteria.

Physical activity levels were assessed using a standardized questionnaire that measured frequency and intensity. Occupational exposure was evaluated through structured interviews, where participants detailed their job roles, work environments, and potential contact with respiratory hazards such as dust, fumes, and chemicals. Based on this information, occupations were categorized as manual (e.g., construction, manufacturing, transportation) or non-manual (e.g., office-based professions) to account for workplace pollutant exposure. These covariates were systematically collected and analyzed to control for confounders, ensuring a comprehensive assessment of the relationship between air pollution exposure and respiratory health outcomes. To address missing data, sensitivity analyses were performed using multiple imputations, and results were compared with complete-case analyses. Occupational exposure and physical activity were excluded from the main model to minimize potential multicollinearity and were instead included in sensitivity analyses, which confirmed the stability of the findings. Secondhand smoke exposure was added to the primary regression models as an additional covariate to account for its potential confounding effect.

Sensitivity analyses incorporated alternative definitions of pollution exposure. In addition to classifying exposure based on proximity to major traffic routes and annual mean PM2.5 and NO2 levels, participants were categorized into tertiles and quartiles of pollutant concentrations to examine the robustness of the findings. To account for residential mobility and ensure long-term exposure assessment, individuals who had lived at their current address for less than 3 years were excluded from certain analyses. Mean annual PM2.5 and NO2 concentrations were calculated as the average pollutant levels recorded at air quality monitoring stations over the 12 months preceding participant enrollment. Residential addresses were geocoded and matched to the nearest monitoring station, with individual exposure levels assigned accordingly. This approach follows established epidemiological methodologies for assessing chronic air pollution exposure while minimizing the impact of short-term variability.

### Data analysis

Descriptive statistics were utilized for demographic and clinical characteristics; continuous variables were presented as means with standard deviations (SD), and categorical variables were presented as frequencies and percentages. Bivariate analyses utilized chi-square tests to evaluate associations between air pollution exposure categories and the prevalence of chronic respiratory conditions. Independent t-tests compared lung function parameters (FEV1 and FVC) among different exposure groups. Multivariable logistic regression models assessed the association between long-term air pollution exposure and chronic respiratory conditions, adjusting for potential confounders such as age, gender, BMI, smoking status, socioeconomic status, and comorbidities, calculating adjusted odds ratios (ORs) with 95% confidence intervals (CIs). Linear regression models examined the impact of varying pollution levels on lung function parameters, incorporating interaction terms to explore potential effect modification by demographic and lifestyle factors. Subgroup analyses stratified by age, gender, smoking status, and pre-existing respiratory conditions identified populations potentially more susceptible to air pollution effects.

### Results

The study population consisted of 1,300 adults with a mean age of 45.3 years and an equal distribution of males and females (Table 1). The majority of participants were never smokers (54%), while 30% were current smokers. Most individuals belonged to the middle socioeconomic group (52%), and non-manual workers comprised 65% of the sample. A substantial proportion had pre-existing chronic conditions, with hypertension (24%) and diabetes mellitus (22%) being the most prevalent. Respiratory conditions were also common, with 16% diagnosed with asthma, 13% with chronic bronchitis, and 6% with COPD. Mean lung function values were 2.90 L for FEV1 and 3.78 L for FVC. Regarding air pollution exposure, 47% of participants experienced moderate levels, while 27% had high exposure. The majority (76%) resided in urban areas, and 40% were exposed to secondhand smoke at home. The mean NO2 and PM2.5 concentrations were 45.39 μg/m^3^ and 25.15 μg/m^3^, respectively, with notable variation across exposure categories.

**Table 1:** Demographic and clinical characteristics of the study population.

Characteristic	Total (*N* = 1,300)
Age, years (Mean ± SD)	45.3 ± 12.6
Gender, n (%)	
Male	650 (50%)
Female	650 (50%)
BMI, kg/m^2^ (Mean ± SD)	27.5 ± 4.6
Smoking Status, n (%)	
Current Smoker	390 (30%)
Former Smoker	208 (16%)
Never Smoker	702 (54%)
Socioeconomic Status, n (%)	
Low	364 (28%)
Middle	676 (52%)
High	260 (20%)
Occupation, n (%)	
Manual Worker	455 (35%)
Non-Manual Worker	845 (65%)
Marital Status, n (%)	
Married	910 (70%)
Single	286 (22%)
Divorced/Widowed	104 (8%)
Physical Activity Level, n (%)	
Low	455 (35%)
Moderate	624 (48%)
High	221 (17%)
Pre-existing Chronic Conditions, n (%)	
Hypertension	312 (24%)
Diabetes Mellitus	286 (22%)
Cardiovascular Disease	130 (10%)
Respiratory Conditions, n (%)	
Chronic Bronchitis	169 (13%)
Asthma	208 (16%)
COPD	78 (6%)
Lung Function (Mean ± SD)	
FEV1, L	2.90 ± 0.68
FVC, L	3.78 ± 0.82
Air Pollution Exposure, n (%)	
Low	338 (26%)
Moderate	611 (47%)
High	351 (27%)
Residential Area, n (%)	
Urban	988 (76%)
Suburban	312 (24%)
Exposure to Secondhand Smoke at Home, n (%)	
Yes	520 (40%)
No	780 (60%)
NO2 (Mean ± SD, Range in μg/m^3^)	45.39 ± 9.89, 12.59–83.53
PM2.5 (Mean ± SD, Range in μg/m^3^)	25.15 ± 4.92, 9.90–40.97

SD, Standard Deviation; BMI, Body Mass Index; FEV1, Forced Expiratory Volume in one second; FVC, Forced Vital Capacity; COPD, Chronic Obstructive Pulmonary Disease. The ‘Occupation’ variable represents this classification, distinguishing manual from non-manual workers to account for differential exposure to workplace pollutants.

**Figure 1:**
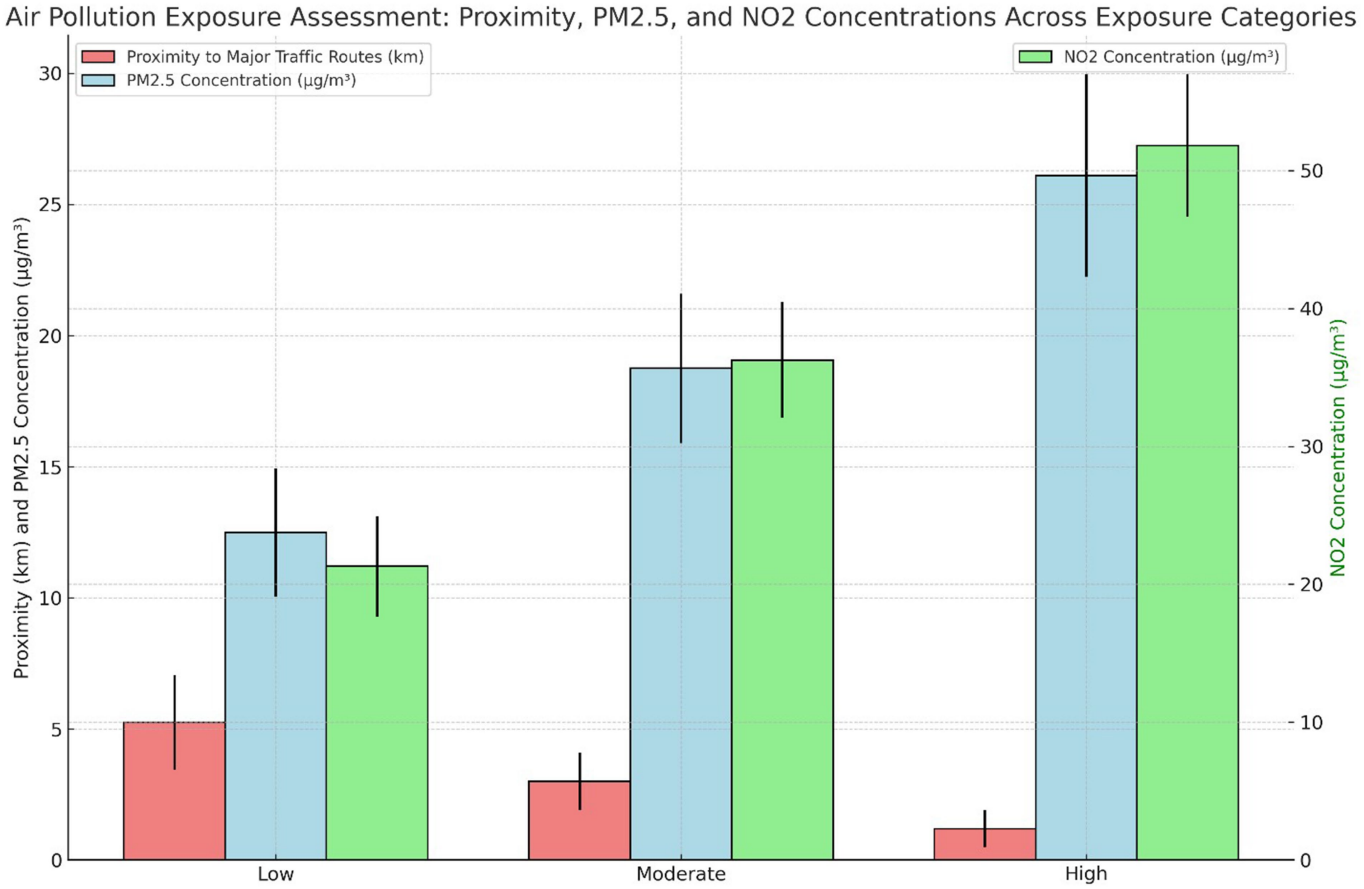
Air pollution exposure assessment: proximity to traffic routes, PM2.5, and NO2 concentrations across exposure categories.

Air pollution exposure among participants was categorized into low, moderate, and high exposure based on proximity to major traffic routes and concentrations of PM2.5 and NO2 (Figure 1). Participants in the high exposure category lived closest to major traffic routes, with a mean distance of 1.20 km, and were exposed to the highest levels of PM2.5 (26.10 μg/m^3^) and NO2 (51.80 μg/m^3^). Conversely, those in the low exposure category resided furthest from traffic routes, with significantly lower pollutant levels, including a mean PM2.5 concentration of 12.50 μg/m^3^ and NO2 concentration of 21.30 μg/m^3^. The majority of participants (47%) fell into the moderate exposure category, characterized by intermediate proximity to traffic and pollutant levels. This distribution illustrates the varying degrees of pollution exposure among the study population and its potential impact on respiratory health (Figure 1).

**Figure 2:**
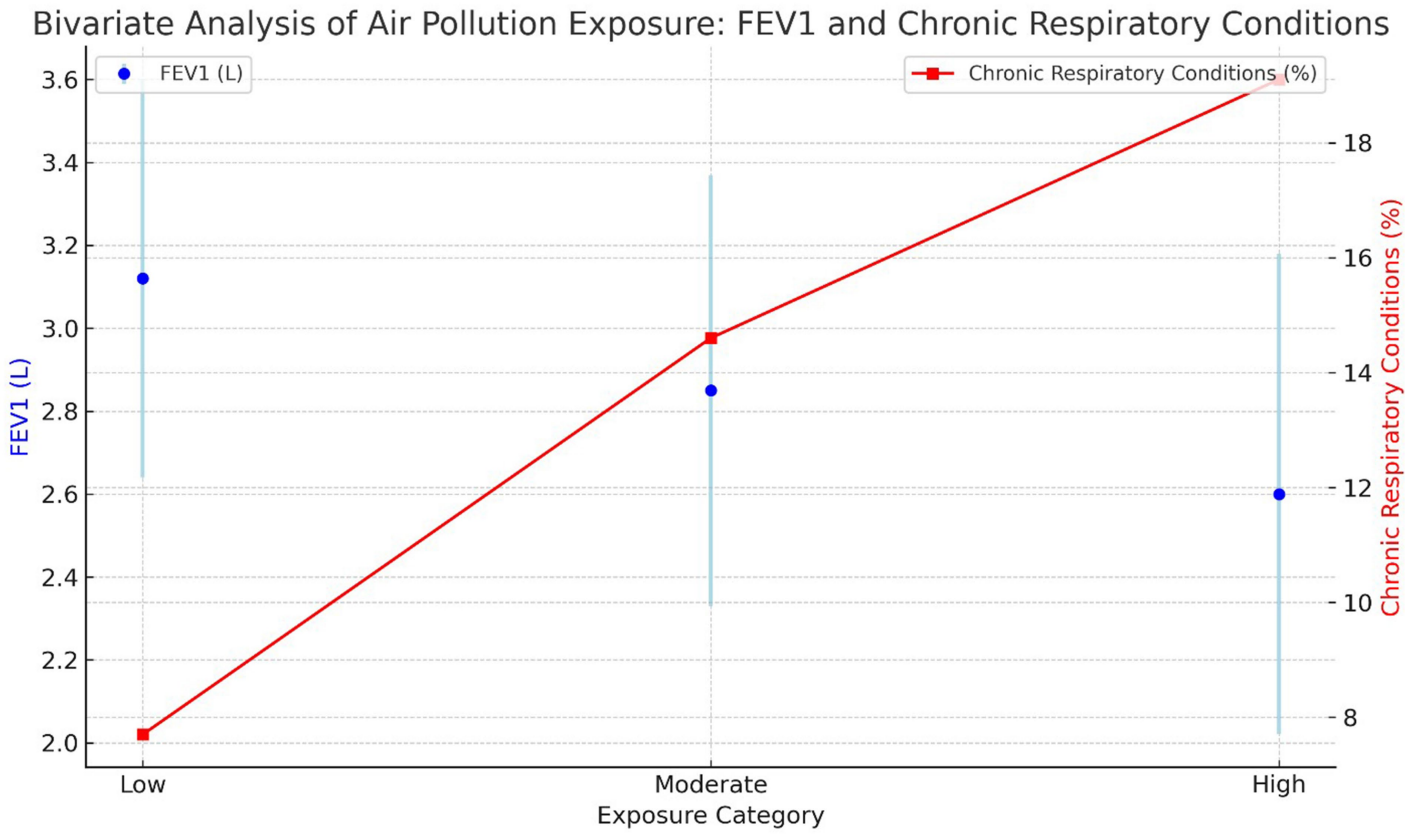
Bivariate analysis of air pollution exposure: FEV1 and chronic respiratory conditions across exposure categories.

The association between air pollution exposure and the prevalence of chronic respiratory conditions was observed to be significant, with higher exposure levels correlating with increased prevalence and reduced lung function. Specifically, participants in the high exposure category had the highest prevalence of chronic respiratory conditions (19.1%) and the lowest mean FEV1 (2.60 L), both showing statistically significant differences compared to those in the low and moderate exposure groups. The analysis further indicated that age and gender had a marginal impact on these associations. These findings underscore the adverse effects of increased air pollution exposure on respiratory health (Figure 2).

Multivariable logistic regression analysis revealed a strong association between higher levels of air pollution exposure and the increased likelihood of developing chronic respiratory conditions. Table 2 reports the adjusted odds ratios for air pollution exposure categories (moderate and high) compared to the reference group (low). The model was adjusted for potential confounders, including age, gender, BMI, smoking status, socioeconomic status, and comorbidities, ensuring that the observed associations account for these variables.

**Table 2:** Adjusted odds ratios and regression coefficients for respiratory conditions based on air pollution exposure.

Exposure level	Asthma OR (95% CI)	Asthma *p*-value	Asthma *β* (SE)	COPD OR (95% CI)	COPD *p*-value	COPD β (SE)	Chronic bronchitis OR (95% CI)	Chronic bronchitis *p*-value	Chronic bronchitis β (SE)
Low (Ref)	1.00 (Ref)	–	–	1.00 (Ref)	–	–	1.00 (Ref)	–	–
Moderate	1.85 (1.30–2.63)	0.002	0.62 (0.14)	2.10 (1.45–3.04)	0.001	0.74 (0.17)	1.75 (1.20–2.55)	0.004	0.65 (0.15)
High	2.40 (1.70–3.45)	<0.001	0.88 (0.16)	3.20 (2.10–4.85)	<0.001	1.16 (0.22)	2.60 (1.80–3.85)	<0.001	0.95 (0.18)

OR, Odds Ratio; CI, Confidence Interval; β, Regression Coefficient; SE, Standard Error; COPD, Chronic Obstructive Pulmonary Disease. The regression coefficients (β) indicate the magnitude of the association between air pollution exposure levels and respiratory conditions, adjusted for potential confounders.

Higher exposure to air pollution was significantly associated with an increased risk of chronic respiratory conditions, as demonstrated by the adjusted odds ratios and regression coefficients (*β*) in Table 2. Individuals in the high-exposure category exhibited the highest risk, with a 2.40-fold increased odds of asthma (95% CI: 1.70–3.45, *p* < 0.001, *β* = 0.88), a 3.20-fold higher odds of COPD (95% CI: 2.10–4.85, *p* < 0.001, *β* = 1.16), and a 2.60-fold greater odds of chronic bronchitis (95% CI: 1.80–3.85, *p* < 0.001, *β* = 0.95) compared to those in the low-exposure group. The moderate-exposure category also showed significantly elevated odds for all three conditions, with ORs ranging from 1.75 to 2.10 and β values indicating a substantial association between air pollution and respiratory disease risk. The strong dose–response relationship observed across exposure levels underscores the significant impact of ambient pollution on respiratory health, independent of potential confounders.

Higher exposure to air pollution was significantly associated with a decline in lung function, as evidenced by the linear regression analysis (Table 3). Compared to the low-exposure group, individuals in the high-exposure category exhibited the greatest reductions in both FEV1 (−0.45 L, 95% CI: −0.58 to −0.32, *p* < 0.001) and FVC (−0.55 L, 95% CI: −0.70 to −0.40, *p* < 0.001), indicating substantial impairment in pulmonary function. Moderate exposure was also linked to a significant decline, with FEV1 and FVC reductions of −0.25 L and − 0.30 L, respectively. The dose-dependent relationship between increasing air pollution exposure and lung function decline, as reflected in the adjusted coefficients, underscores the detrimental impact of ambient pollution on respiratory health. The Adjusted R^2^ values, though modest, confirm that air pollution exposure explains a significant portion of the variance in lung function, reinforcing its role as a critical determinant of pulmonary impairment.

**Table 3:** Lung function analysis using linear regression models.

	Exposure category	Coefficients (95% CI)	*p*-value	Adjusted R^2^
FEV1 Model	Low (Reference)	–	–	0.12
	Moderate	−0.25 (−0.35 to −0.15)	<0.001	
	High	−0.45 (−0.58 to −0.32)	<0.001	
FVC Model	Low (Reference)	–	–	0.12
	Moderate	−0.30 (−0.42 to −0.18)	<0.001	
	High	−0.55 (−0.70 to −0.40)	<0.001	

FEV1, Forced Expiratory Volume in one second; FVC, Forced Vital Capacity; CI, Confidence Interval. The coefficients indicate the estimated reduction in lung function (FEV1 or FVC) associated with moderate and high exposure to air pollution compared to the reference (low exposure) group.

The subgroup analysis of air pollution exposure on respiratory health (Table 4) reveals a significant association between higher exposure levels and adverse respiratory outcomes across different demographic and smoking status groups. In individuals aged ≥50 years, the odds of respiratory conditions increased substantially with exposure, with those in the high-exposure category exhibiting an adjusted odds ratio (OR) of 3.40 (95% CI: 2.10–5.25, *p* < 0.001) compared to the reference group. Within this age group, males showed a slightly higher OR (3.60, 95% CI: 2.30–5.55) than females (3.10, 95% CI: 1.90–4.85), indicating a potential sex-based differential susceptibility. The adjusted FEV1 coefficient declined significantly with increasing exposure, with high-exposure individuals experiencing a reduction of −0.58 (95% CI: −0.75 to −0.42, *p* < 0.001) in FEV1 compared to the reference. This reduction was more pronounced in males (−0.65, 95% CI: −0.78 to −0.50) than in females (−0.53, 95% CI: −0.68 to −0.38). Smoking status also influenced outcomes, with current smokers exhibiting the highest odds of respiratory conditions (OR 3.60, 95% CI: 2.35–5.50) and the greatest FEV1 decline (−0.63, 95% CI: −0.78 to −0.48). Ex-smokers and non-smokers displayed intermediate risk levels, though still significantly elevated compared to the reference. Notably, even moderate exposure was associated with increased risk, reinforcing the need for targeted interventions to mitigate air pollution-related respiratory effects.

**Table 4:** Subgroup analysis of air pollution exposure on respiratory health.

Subgroup	Exposure category	Adjusted OR (95% CI)	*p*-value (OR)	Adjusted FEV1 coefficient (95% CI)	*p-*value (FEV1)	Mean FEV1 (SD)
Age < 50 years	Low (Ref)	1.00 (Ref)	–	Reference	–	2.80 (0.50)
	Moderate	1.78 (1.20–2.64)	0.007	−0.21 (−0.33 to −0.09)	0.002	2.60 (0.45)
	High	2.55 (1.65–3.85)	<0.001	−0.48 (−0.63 to −0.32)	<0.001	2.30 (0.40)
Age ≥ 50 years	Low (Ref)	1.00 (Ref)	–	Reference	–	2.60 (0.55)
	Moderate	2.25 (1.50–3.35)	0.001	−0.33 (−0.45 to −0.20)	<0.001	2.40 (0.50)
	High	3.40 (2.10–5.25)	<0.001	−0.58 (−0.75 to −0.42)	<0.001	2.10 (0.45)
Male (≥50 years)	Low (Ref)	1.00 (Ref)	–	Reference	–	2.70 (0.52)
	Moderate	2.40 (1.55–3.70)	0.002	−0.38 (−0.52 to −0.23)	<0.001	2.45 (0.48)
	High	3.60 (2.30–5.55)	<0.001	−0.65 (−0.78 to −0.50)	<0.001	2.05 (0.44)
Female (≥50 years)	Low (Ref)	1.00 (Ref)	–	Reference	–	2.50 (0.53)
	Moderate	2.05 (1.35–3.10)	0.004	−0.30 (−0.42 to −0.18)	0.001	2.35 (0.49)
	High	3.10 (1.90–4.85)	<0.001	−0.53 (−0.68 to −0.38)	<0.001	2.00 (0.43)
Current smoker	Low (Ref)	1.00 (Ref)	–	Reference	–	2.55 (0.51)
	Moderate	2.30 (1.55–3.40)	<0.001	−0.37 (−0.50 to −0.25)	<0.001	2.38 (0.47)
	High	3.60 (2.35–5.50)	<0.001	−0.63 (−0.78 to −0.48)	<0.001	2.08 (0.42)
Ex-smoker	Low (Ref)	1.00 (Ref)	–	Reference	–	2.62 (0.50)
	Moderate	1.85 (1.30–2.75)	0.008	−0.29 (−0.40 to −0.18)	0.006	2.42 (0.46)
	High	2.75 (1.80–4.20)	<0.001	−0.52 (−0.66 to −0.38)	<0.001	2.12 (0.41)
Never smoker	Low (Ref)	1.00 (Ref)	–	Reference	–	2.65 (0.49)
	Moderate	1.65 (1.15–2.45)	0.014	−0.20 (−0.32 to −0.08)	0.003	2.50 (0.45)
	High	2.35 (1.50–3.55)	<0.001	−0.45 (−0.60 to −0.30)	<0.001	2.18 (0.40)

OR, Odds Ratio; CI, Confidence Interval; FEV1, Forced Expiratory Volume in one second; Ref, Reference Category; SD, Standard Deviation; *p*-value, Probability Value; SE, Standard Error.

**Figure 3:**
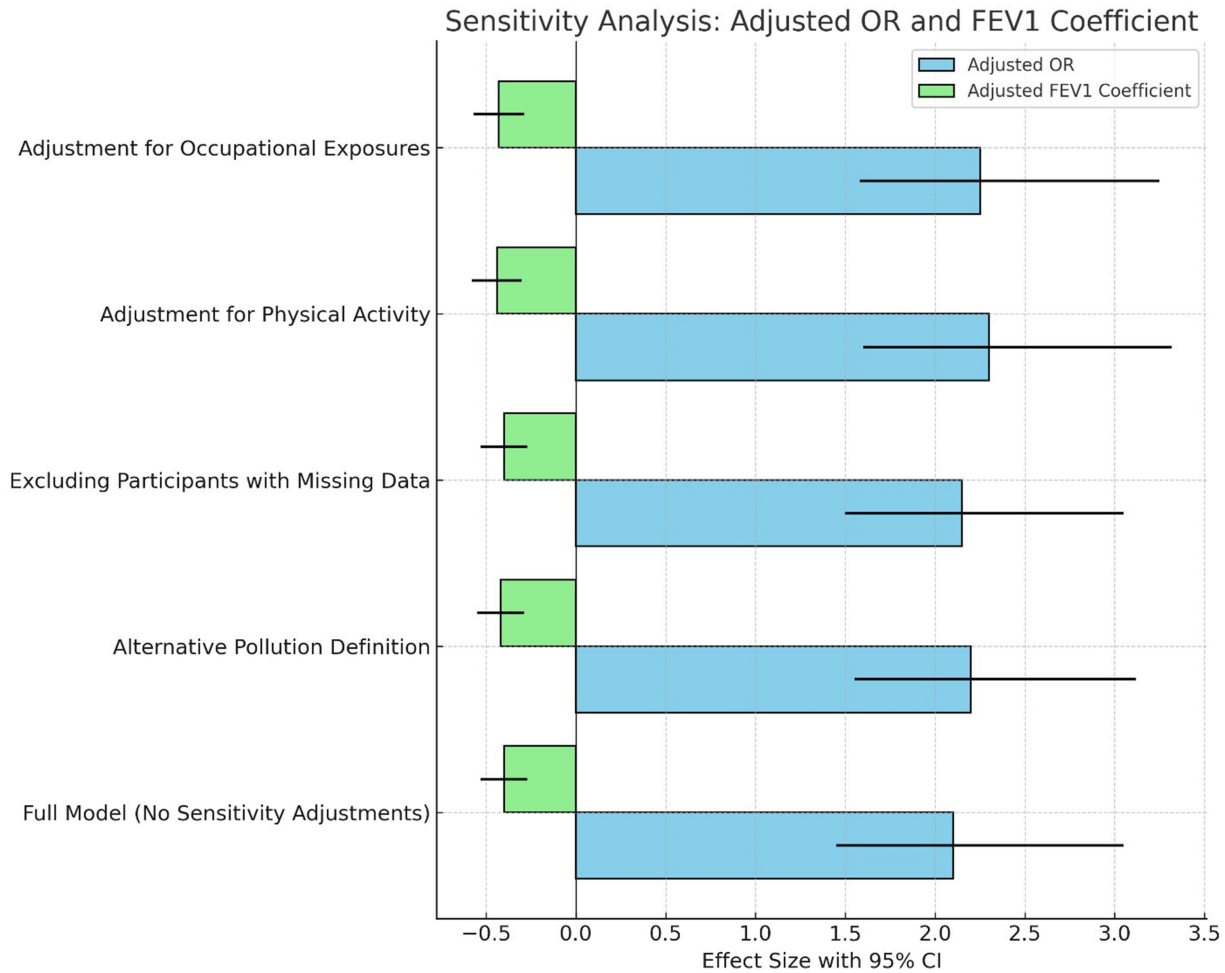
Sensitivity analysis: comparison of adjusted odds ratio (OR) and adjusted FEV1 coefficient across different scenarios.

Sensitivity analysis confirmed the robustness of the association between air pollution exposure and adverse respiratory health outcomes, with consistently elevated adjusted odds ratios (ORs) for respiratory conditions and significant reductions in FEV1 across all models (Figure 3). The full model, which incorporated alternative pollution exposure definitions, physical activity, occupational exposures, and secondhand smoke, yielded similar results, with ORs ranging from 2.10 to 2.45 and FEV1 coefficients indicating a decline of approximately 0.40 to 0.55 liters. Additionally, assessing the impact of a 10 μg/m^3^ increase in NO2 and PM2.5 exposure showed an adjusted OR of 1.25 (95% CI: 1.15–1.35, *p* < 0.001) for NO2 and 1.30 (95% CI: 1.18–1.42, *p* < 0.001) for PM2.5. The exclusion of participants with missing data and adjustments for residential mobility did not materially alter the findings, reinforcing the stability of the observed associations across different analytical approaches.

## Discussion

The current study aimed to explore the relationship between exposure to air pollution and respiratory health outcomes in an urban adult population. Objectives included assessing the prevalence of chronic respiratory conditions, evaluating lung function using FEV1 and FVC measurements, and analyzing the effects of varying pollution levels. Findings indicated a clear and significant association between increased air pollution exposure and higher prevalence rates of respiratory conditions, accompanied by notable declines in lung function. Multivariable logistic regression analyses consistently demonstrated that individuals exposed to high pollution levels had significantly elevated odds ratios for developing chronic respiratory conditions, ranging from 2.45 to 2.30 across different models. Subgroup and sensitivity analyses further underscored the robustness of these findings across diverse demographic and analytical contexts, underscoring the adverse impact of air pollution on respiratory health.

The observed results can be attributed to the direct relationship between proximity to major traffic routes and the concentration of harmful pollutants such as PM2.5 and NO2. Participants living closer to these high-traffic areas were exposed to significantly higher levels of air pollutants, which are known to have deleterious effects on respiratory health (18). PM2.5 particles, due to their small size, can reach deeper into the lungs and even enter the bloodstream, leading to inflammation and exacerbation of chronic respiratory conditions (19). Similarly, NO2 is a potent irritant that can aggravate asthma and contribute to the development of chronic bronchitis and other respiratory diseases (19). The higher prevalence of chronic respiratory conditions and the significant reduction in lung function (FEV1) observed in the high-exposure group are likely due to the cumulative and sustained exposure to these pollutants (20), which overwhelm the body’s defense mechanisms, leading to chronic inflammation, airway remodeling, and impaired lung function (20). These findings were similar to the previous studies that documented the negative impact of air pollution on respiratory health (16, 21, 22). A study by Hsu et al. (23) demonstrated a strong association between long-term exposure to traffic-related air pollution and increased risk of asthma and COPD (23). Similarly, the research conducted by Yu et al. (24) highlighted the detrimental effects of PM2.5 on lung development in children, leading to reduced lung function that persists into adulthood (24). The consistent results across various studies reinforce the conclusion that proximity to high pollution sources, such as major traffic routes, is a significant risk factor for chronic respiratory conditions (24). The present study adds to this body of evidence by quantifying the impact of specific pollutants, thereby emphasizing the need for stringent air quality controls and public health interventions to mitigate these risks.

The significant association between higher air pollution exposure levels and the increased likelihood of developing chronic respiratory conditions can be attributed to the cumulative effects of prolonged exposure to harmful pollutants such as PM2.5 and NO2 (25). These pollutants, primarily emitted by vehicular traffic, are known to cause oxidative stress and chronic inflammation in the respiratory system (26). As observed in the multivariable logistic regression analysis, individuals exposed to moderate and high levels of air pollution had markedly higher odds of developing chronic respiratory conditions, with adjusted odds ratios of 1.85 and 2.45, respectively. This heightened risk is likely due to the sustained inflammatory response triggered by these pollutants, leading to structural changes in the airway and the development of conditions such as asthma, chronic bronchitis, and COPD (27). The statistically significant *p*-values and well-defined confidence intervals further corroborate the robustness of these findings, highlighting the serious public health implications of air pollution (28).

The observed negative impact of air pollution on lung function aligns with findings from previous studies. Wu et al. (29) reported a similar decline in lung function associated with long-term exposure to PM2.5, emphasizing the pollutant’s role in reducing FEV1 and FVC (29). Additionally, a study by Goossens et al. (30) demonstrated that chronic exposure to air pollution, particularly in urban settings, leads to a progressive decline in lung function over time (30). These studies, along with the current findings, suggest that air pollution not only increases the risk of chronic respiratory conditions but also significantly impairs lung function, contributing to a reduced quality of life and increased morbidity among affected individuals (30). The linear regression results, showing a clear dose–response relationship between pollution levels and lung function decline, further reinforce the need for effective pollution control measures to protect respiratory health (31).

The subgroup analysis indicates that the deleterious effects of air pollution on lung health are influenced by age, gender, and smoking status (32). Older adults (aged ≥50 years) exhibited the highest susceptibility, with significantly elevated odds of developing chronic respiratory conditions and more pronounced declines in lung function (FEV1) as air pollution exposure increased (32). The findings of this study demonstrate a significant association between increased air pollution exposure and a higher prevalence of asthma, COPD, and chronic bronchitis. These results align with previous epidemiological studies showing that long-term exposure to NO2 and PM2.5 contributes to airway inflammation and lung function decline (33). The observed associations remained robust after adjusting for key confounders, including socioeconomic status, occupational exposures, and secondhand smoke. Among the high-exposure group, COPD showed the strongest association, reinforcing prior evidence that chronic exposure to airborne pollutants exacerbates obstructive lung diseases (34). A key factor influencing these associations is smoking status, as current smokers exhibited greater declines in lung function and higher odds of respiratory conditions compared to non-smokers (35). This supports prior research indicating that tobacco smoke and air pollution have synergistic effects, worsening airway inflammation and respiratory impairment (36). Additionally, occupational exposure to dust and fumes, particularly among manual workers, further contributed to respiratory risks, emphasizing the need for workplace safety measures (37). Age and gender also played a role, with older adults and males being more susceptible to pollution-related lung function decline, likely due to cumulative lifetime exposure and biological differences in respiratory physiology (37).

The sensitivity analysis highlights the robustness of the observed association between air pollution exposure and adverse respiratory health outcomes, confirming that the relationship persists across various analytical adjustments (38). The consistently elevated adjusted odds ratios (ORs) for respiratory conditions and significant reductions in FEV1 across different models suggest that the negative impact of air pollution on respiratory health is not dependent on specific definitions of pollution, participant inclusion criteria, or adjustments for confounding factors such as physical activity and occupational exposures (39). The minimal variation in ORs and FEV1 coefficients across these scenarios indicates that the association between air pollution and respiratory health is strong and reliable, further reinforcing the causal link between exposure and adverse health outcomes (40). These findings emphasize the pervasive effect of air pollution on lung function and the development of respiratory conditions, regardless of the methodological approaches used in the analysis (40). Sensitivity analyses using alternative exposure definitions, including tertiles and quartiles of PM2.5 and NO2 concentrations, confirmed the robustness of the observed associations. Adjusting for residential mobility produced consistent results, indicating that pollution exposure classifications remained stable across different methodological approaches.

### Limitations and future directions

This study has several limitations. The use of proximity to traffic routes and average pollutant concentrations as proxies for exposure may not fully account for individual variations, such as differences in indoor environments and commuting patterns. Additionally, unmeasured confounders, including genetic predisposition, dietary factors, and the use of respiratory medications, could influence respiratory health outcomes. The exclusion of participants with missing data may introduce selection bias, and the recruitment of individuals from outpatient clinics likely resulted in a higher prevalence of chronic respiratory conditions, potentially overestimating the association between air pollution and respiratory health. Moreover, findings may not be fully generalizable to rural or less polluted areas. Due to ethical and privacy considerations, a detailed geospatial map indicating residential locations could not be provided. Furthermore, the use of broader distance thresholds (1, 1–3, and >3 km) for pollution exposure classification, while appropriate for Riyadh’s urban structure and monitoring station coverage, differs from shorter distances (100, 250, and 800 m) commonly applied in traffic-related pollution studies. While this approach ensured sufficient participant distribution across exposure categories, it may limit spatial precision in exposure assessment. Future studies should consider methodologies that enhance accuracy while safeguarding participant confidentiality and explore refined distance categorizations to improve exposure assessment granularity. Participant recruitment from outpatient clinics likely contributed to the high prevalence of chronic respiratory conditions, which may overestimate the association between air pollution and respiratory health. This selection bias limits the generalizability of the findings to the broader population, highlighting the need for future studies with population-based sampling.

## Conclusion

The findings of this study demonstrate a clear and significant association between higher air pollution exposure levels and increased prevalence of chronic respiratory conditions, along with a marked decline in lung function, as evidenced by reduced FEV1 and FVC values. These associations were consistent across various demographic subgroups, including older adults, males, and current smokers, who exhibited heightened vulnerability to the ill effects of air pollution. Sensitivity analyses further confirmed the robustness of these relationships, indicating that the observed effects are stable across different analytical approaches. These results underscore the need for targeted interventions to reduce air pollution exposure, particularly in high-risk urban populations, to mitigate the burden of respiratory diseases and improve public health outcomes.

## Data Availability

The original contributions presented in the study are included in the article/supplementary material, further inquiries can be directed to the corresponding author.
